# A complex gene locus enables xyloglucan utilization in the model saprophyte *Cellvibrio japonicus*

**DOI:** 10.1111/mmi.12776

**Published:** 2014-09-17

**Authors:** Johan Larsbrink, Andrew J Thompson, Magnus Lundqvist, Jeffrey G Gardner, Gideon J Davies, Harry Brumer

**Affiliations:** 1 Division of Glycoscience, School of Biotechnology, Royal Institute of Technology (KTH), AlbaNova University Centre106 91, Stockholm, Sweden; 2 Department of Chemistry, University of YorkHeslington, York, YO10 5DD, UK; 3 Department of Biological Sciences, University of Maryland – Baltimore County1000 Hilltop Circle, Baltimore, MD, 21250, USA; 4 Michael Smith Laboratories and Department of Chemistry, University of British Columbia2185 East Mall, Vancouver, BC, V6T 1Z4, Canada

## Abstract

The degradation of plant biomass by saprophytes is an ecologically important part of the global carbon cycle, which has also inspired a vast diversity of industrial enzyme applications. The xyloglucans (XyGs) constitute a family of ubiquitous and abundant plant cell wall polysaccharides, yet the enzymology of XyG saccharification is poorly studied. Here, we present the identification and molecular characterization of a complex genetic locus that is required for xyloglucan utilization by the model saprophyte *Cellvibrio japonicus*. In harness, transcriptomics, reverse genetics, enzyme kinetics, and structural biology indicate that the encoded cohort of an α-xylosidase, a β-galactosidase, and an α-l-fucosidase is specifically adapted for efficient, concerted saccharification of dicot (fucogalacto)xyloglucan oligosaccharides following import into the periplasm via an associated TonB-dependent receptor. The data support a biological model of xyloglucan degradation by *C. japonicus* with striking similarities – and notable differences – to the complex polysaccharide utilization loci of the Bacteroidetes.

## Introduction

The saccharification of diverse types of plant biomass is both an ecologically important part of the global carbon cycle and a biotechnologically relevant aspect of the food, feed, biofuel, biomaterials, and cleaning-product industries. Hydrolysis of the complex plant cell wall to acquire sugars for growth presents a significant challenge for microorganisms, and requires a wide range of enzyme activities to address the tremendous diversity of glycosidic, peptide, polyphenolic, and ester linkages present (Carpita and McCann, 2000; Jovanovic *et al.*, 2009). Whereas the complete hydrolysis of cellulose microfibres is difficult due to their semi-crystalline nature, the monosaccharide and linkage complexity of the hemicelluloses and pectins additionally confounds enzymatic attack. Moreover, the intertwining of plant cell wall biomolecules into a composite material provides an additional level of structural complexity mitigating biomass saccharification, and there is growing evidence that addition of hemicellulases to industrial enzyme cocktails can improve efficacy (Hu *et al.*, 2013; Jabbour *et al.*, 2013). As such, carbohydrate-active enzyme (CAZyme) discovery remains a vibrant area of research (Jovanovic *et al.*, 2009; Mewis *et al.*, 2013; Hemsworth *et al.*, 2014; del Pulgar and Saadeddin, 2014).

*Cellvibrio japonicus* (previously *Pseudomonas fluorescens* subsp. *cellulosa*) is a Gram-negative bacterium that was first isolated from Japanese soil in the 1950s and has since become intensely studied due to its ability to degrade all common plant cell wall polysaccharides (Hazlewood and Gilbert, 1998; Deboy *et al.*, 2008). As a model saprophytic organism, the complete genome sequence of *C. japonicus* has been determined and genetic tools have been established (Deboy *et al.*, 2008; Gardner and Keating, 2012). *C. japonicus* is thus important both for CAZyme discovery and for fundamental molecular studies of polysaccharide degradation by Gram-negative environmental bacteria. Furthermore, recent metabolic engineering of a strain with the ability to produce ethanol has demonstrated the potential of *C. japonicus* for industrial chemical production in consolidated bioprocessing (Gardner and Keating, 2010). Whereas the mannan-, xylan-, and arabinan-degrading systems have been intensively studied (Cartmell *et al.*, 2008; 2011,; Deboy *et al.*, 2008; Emami *et al.*, 2009 and references therein], the capacity of *C. japonicus* to degrade the ubiquitous hemicellulose family of xyloglucans has received little attention.

The xyloglucans (XyG) are a structurally complex family of polysaccharides found in all terrestrial plants (Carpita and McCann, 2000; Popper *et al.*, 2011). In dicots alone, XyGs may account for 20–25% of primary cell wall dry weight (Scheller and Ulvskov, 2010) and, as such, constitute an important terrestrial carbon sink. XyGs are composed of a cellulose-like linear β(1→4)-glucan backbone appended with branching α(1→6)-xylosyl moieties, which can in turn be extended with additional glycosides (e.g. galacto-, fuco- and arabinosyl moieties) in a tissue- and species-dependent manner (Peña *et al.*, 2008; Tuomivaara *et al.*, 2014). Thus, the complete saccharification of XyGs necessitates a cohort of enzymes to address the linkage diversity present in individual XyG variants. Aligned with our continuing interest in xyloglucan-active enzymes (Gilbert *et al.*, 2008; Ariza *et al.*, 2011; Mark *et al.*, 2011; Eklöf *et al.*, 2012; 2013,; Larsbrink *et al.*, 2014), we previously reported the biochemical and structural characterization of the main α-xylosidase of *C. japonicus*, *Cj*Xyl31A, which specifically hydrolyses terminal non-reducing-end xylose moieties of xyloglucan oligosaccharides (XyGOs) (Larsbrink *et al.*, 2011). In the same study, we also identified that *C. japonicus* produces at least one extracellular *endo*-xyloglucanase capable of generating XyGOs by xyloglucan backbone cleavage. However, a key remaining question is, which are the other players in *C. japonicus* that might work in concert with these enzymes to fully deconstruct complex xyloglucans?

**Figure 1:**
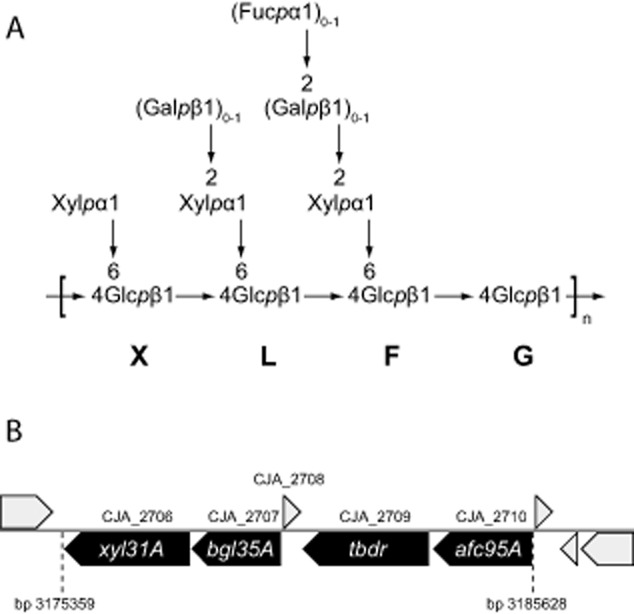
Xyloglucan structure and the arrangement of xyloglucan-degrading genes within the *C. japonicus* genome. A. Typical (fucogalacto)xyloglucan structure, with abbreviated motif nomenclature according to Tuomivaara *et al.* (2014) indicated. B. The xyloglucan utilization locus (XyGUL) in the genome of *C. japonicus*; hypothetical proteins are shown in grey.

Here, we report the identification and molecular characterization of a unique xyloglucan utilization locus (XyGUL) in the genome of *C. japonicus* (Fig. 1). In addition to the α-xylosidase *Cj*Xyl31A, this locus contains a predicted β-galactosidase, a predicted α-l-fucosidase, and a predicted TonB-dependent receptor (Ferguson and Deisenhofer, 2002; Koebnik, 2005), whose collective presence indicates a role in dicot (fucogalacto)xyloglucan degradation and uptake. Using a combination of transcriptional analysis, reverse genetics, subcellular protein localization, recombinant enzyme kinetics, and structural biology, we were able to verify these predictions of glycoside function and extend a biological model of XyG utilization by this important saprophyte.

## Results

### Genome walking reveals a putative xyloglucan utilization locus (XyGUL) in *C. japonicus*

Genome walking *in silico* revealed the presence of open reading frames encoding a predicted β-galactosidase from glycoside hydrolase (GH) family 35 (*Cj*Bgl35A, encoded by locus tag CJA_2707, *bgl35A*), a predicted TonB-dependent receptor (TBDR, encoded by CJA_2709), and a predicted α-l-fucosidase from glycoside hydrolase family 95 (*Cj*Afc95A, encoded by CJA_2710, *afc95A*) upstream from the gene encoding the known α-xylosidase *Cj*Xyl31A (CJA_2706, *xyl31A*) (Fig. 1). Protein and gene names used here correspond to those proposed in Table S2 of Deboy *et al.* (2008). The predicted protein sequences of both *Cj*Bgl35A and *Cj*Afc95A were compared to previously characterized enzymes in GH35 and GH95, respectively, using blast. Interestingly, *Cj*Bgl35A showed a high homology only to the *Xanthomonas campestris* pv. *campestris* GalD β-galactosidase, with a protein identity of 55% and similarity of 70% (over 536 residues), while similarity to other characterized GH35 enzymes catalogued in the CAZy database (Lombard *et al.*, 2014; http://www.cazy.org/GH35_characterized.html) was poor. Moreover, the β-galactosidase activity of GalD has been indicated primarily through genetic studies and minimal specificity data exists for this enzyme (Yang *et al.*, 2007), such that no reliable prediction of substrate specificity for *Cj*Bgl35A can be made. *Cj*Afc95A, in contrast, had high similarity to all five characterized α(1→2)-l-fucosidases of GH95 (Lombard *et al.*, 2014; http://www.cazy.org/GH95_characterized.html), with identities of 31–35% and similarities of 47–52%. In particular, the two plant enzymes (from *Arabidopsis thaliana* and *Lilium longiflorum*) and the fungal enzyme (from *Aspergillus nidulans*) have been shown to cleave the α(1→2)-fucosyl residues from XyGOs (Bauer *et al.*, 2006; Ishimizu *et al.*, 2007; Nagae *et al.*, 2007; Leonard *et al.*, 2008). On the basis of these bioinformatics analyses, we hypothesized that the GHs were involved in the cleavage of β(1→2)-galactosyl and α(1→2)-l-fucosyl residues of dicot (fucogalacto)xyloglucan, respectively, while the TBDR may function in carbohydrate import (Dejean *et al.*, 2013).

### *Cj*Bgl35A and *Cj*Afc95A are XyG-specific glycosidases

To test predictions of the catalytic specificity of the GHs, we performed initial substrate screens on aryl glycosides (Glc*p*/Gal*p*/Xyl*p*-β-PNP, l-Ara*f*-α-PNP and l-Fuc-α-CNP) with both enzymes produced recombinantly in *Escherichia coli*. To further investigate the potential roles of the enzymes *in vivo*, xyloglucan polysaccharides (XyG) and oligosaccharides (XyGOs) from tamarind and iceberg lettuce were examined as natural substrates, including product analysis by HPAEC-PAD and MALDI-TOF. Full kinetic data for both enzymes are listed in Table 1.

**Table 1:** Activity of *Cj*Bgl35A and *Cj*Afc95A on various substrates

Enzyme	Substrate	*k_cat_* (s^−1^)	*K_m_* (mM)	*k_cat_*/*K_m_* (s^−1^ mM^−1^)
*Cj*Bgl35A	Gal-β-PNP	10.1 ± 0.2	0.81 ± 0.05	12.5
	XLLG			3.8 ± 0.08
	Tamarind xyloglucan^a^			0.0031 ± (6.5 × 10^−5^)
*Cj*Afc95A	l-Fuc-α-CNP	26.5 ± 1.1	2.1 ± 0.2	12.9
	XLFG	19.2 ± 0.5	0.21 ± 0.017	89.8
	Lettuce xyloglucan^b^	1.85 ± 0.084	0.26 ± 0.03	7.1

a Kinetic parameters calculated from the available galactosyl moieties of the substrate (16%) as reported by the manufacturer.

b Kinetic parameters calculated from the available fucosyl moieties of the substrate, as determined by end-point hydrolysis by *Cj*Afc95A.

#### *Cj*Bgl35A

*Cj*Bgl35A was able to hydrolyse Gal-β-PNP but did not act upon any of the other substrates in the initial screen. The pH optimum of the enzyme was determined to be pH 5.5 in acetate buffer (Supplemental Fig. S1), but the pH-rate profile deviated from the bell-shaped profile expected for an enzyme mechanism involving two ionizing residues. The *k_cat_/K_m_* value for the reaction on Gal-β-PNP was 12.5 s^−1^ mM^−1^, which is in the same range as another bacterial GH35 β-galactosidase, BgaC from *Streptococcus pneumonia* (49.3 s^−1^ mM^−1^) and the *A. thaliana* BGAL4 (19.1 s^−1^ mM^−1^) (Ahn *et al.*, 2007; Cheng *et al.*, 2012). Hydrolysis of the substrate lactose, which is the preferred substrate for many enzymes in GH35, could not be observed; *Cj*Bgl35A is thus likely not able to generally address β(1→4)-linkages.

In contrast, HPAEC-PAD indicated that the enzyme released galactose from both tamarind seed (galacto)xyloglucan and the doubly galactosylated XLLG nonasaccharide (see Fig. 1 for XyGO nomenclature). Enzyme saturation could not be reached for either substrate (Supplemental Fig. S2). Notably, a nasturtium (*Tropaeolum majus*) β-galactosidase also exhibited a strictly linear dependence of rate on substrate concentration using either nasturtium or tamarind xyloglucan as substrates (Edwards *et al.*, 1988) (it is likely, but not conclusive, that this is a GH35 enzyme: GenBank CAW88932). Apparent *k_cat_/K_m_* values were nonetheless calculated by linear regression of the initial velocities versus substrate concentration plot. Notably, the apparent *k_cat_/K_m_* value for the degalactosylation of tamarind xyloglucan is *c*. 1000-fold lower than that for XLLG (Table 1). This difference, estimated from the total galactose content of the polysaccharide, is likely to represent a lower limit, given the possibility of contaminating galactosylated oligosaccharides (acting as alternate substrates) below our limits of detection and the clear preference of *Cj*Bgl35A for one of the two galactosyl residues (*vide infra*). Regardless, the data indicate that the galactosidase is most likely to act subsequent to the hydrolysis of xyloglucan polysaccharide chains into component oligosaccharides.

**Figure 2:**
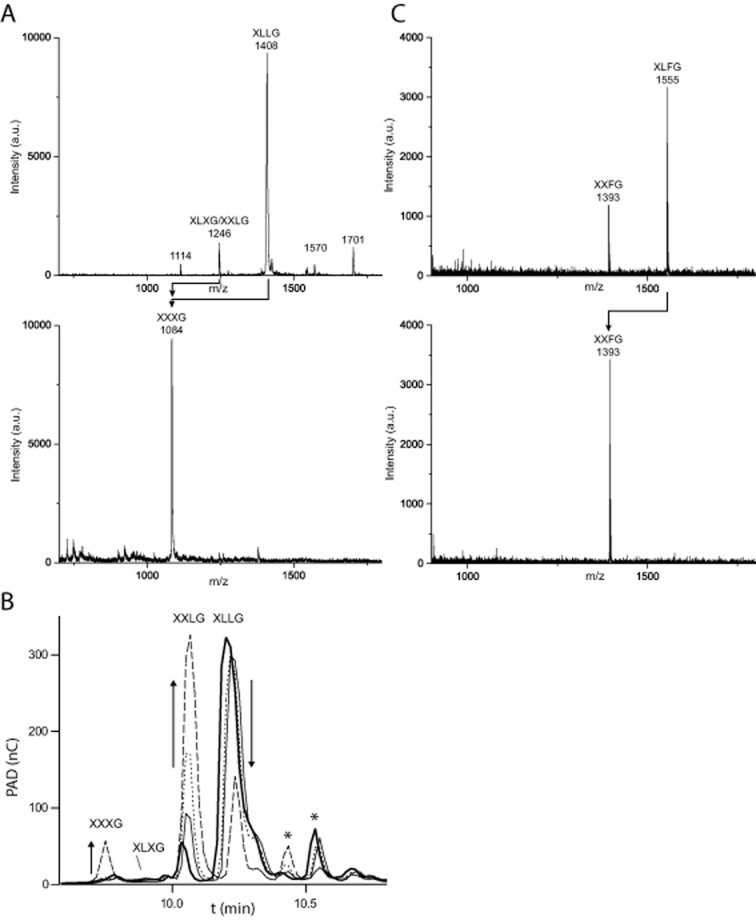
Analysis of products from *Cj*Bgl35A activity on galactosylated xyloglucan oligosaccharides. A. MALDI-TOF spectra of a preparation of XLLG containing a minor amount of XXLG/XLXG before and after incubation with *Cj*Bgl35A, indicating complete hydrolysis of both galactosyl residues. Observed *m/z* values are consistent with [M + Na]^+^ adducts. Minor peaks at *m/z* 1570 and 1701 correspond to unidentified Hex_7_Pen_3_ and Hex_7_Pen_4_ oligosaccharides (Tuomivaara *et al.*, 2014). B. HPAEC-PAD chromatograms of a time-course study of the conversion of XLLG into XXXG via XXLG as a primary product. Arrows indicate trends in peak intensity changes for the starting material and major products. XLLG (bold line) was incubated with *Cj*Bgl35A and samples were analysed after 5 min (thin line), 20 min (dotted line) and 2 h (dashed line). Formation of XLXG is not observed, while XXXG appears only after the majority of the XLLG has been converted into XXLG, revealing that the galactosyl moiety closest to the non-reducing end (cf. Fig. 1) is the preferred substrate for the enzyme. Asterisks indicate minor components, which may correspond to unidentified oligosaccharides observed by MALDI-TOF MS. C. MALDI-TOF spectra of the hydrolysis of a preparation containing XLFG and XXFG by *Cj*Bgl35A. XLFG is completely converted into XXFG while the ‘F’ unit is unchanged, demonstrating that fucosylation blocks the strictly *exo*-acting galactosidase. Observed *m/z* values are consistent with [M+Na]^+^ adducts.

Product analysis by MALDI-TOF MS showed that *Cj*Bgl35A was able to hydrolyse both galactosyl moieties from XLLG to form XXXG (Fig. 2A). A time-course study using HPAEC-PAD revealed an order of preference for the galactosyl residues on the two L (Gal–Xyl–Glc) moieties: XXLG was produced essentially exclusively during the initial stages of the reaction, with XXXG appearing only when a majority of the XLLG had been converted into XXLG. Given this preference, the initial-rate the kinetic data (Table 1, Supplemental Fig. S2) likely reflect the hydrolysis of the galactosyl moiety most distal from the reducing-end (underlined: XLLG). To assess whether the enzyme could cleave galactosyl moieties underlying a terminal fucosyl moiety, a 1:2 mixture of the oligosaccharides XXFG and XLFG (Fig. 1A) was prepared from fucosylated XyG extracted from iceberg lettuce by digestion with a *Bacteroides ovatus* GH5 *endo*-xyloglucanase (Larsbrink *et al.*, 2014) and size-exclusion chromatography. Extended incubation of a high concentration of *Cj*Bgl35A with this mixture (10 μM enzyme, *c.* 0.5 mM substrate, 16 h, 25°C) followed by MALDI-TOF MS demonstrated that only XLFG was hydrolysed, yielding XXFG, which was not further transformed (Fig. 2C). The data indicate that *Cj*Bgl35A acts strictly as an *exo*-galactosidase that requires terminal galactosyl moieties on XyGOs.

At present, no other bacterial enzymes from GH35 have been assayed for XyG specificity, to our knowledge, so direct comparisons with *Cj*Bgl35A are difficult to make. However, many of these are specific for lactose, a substrate not hydrolysed by *Cj*Bgl35A, which suggests distinct activity profiles. Rather, the specificity of *Cj*Bgl35A is similar to plant β-galactosidases with a general ability to remove side-chain galactosyl units from xyloglucan polysaccharides; the GH35 member from *A. thaliana*, Bgal10 (At5g63810) produces XXLG and XXXG sequentially due to a preference for the underlined side-chain in XLLG (Sampedro *et al.*, 2012 and references therein).

#### *Cj*Afc95A

**Figure 3:**
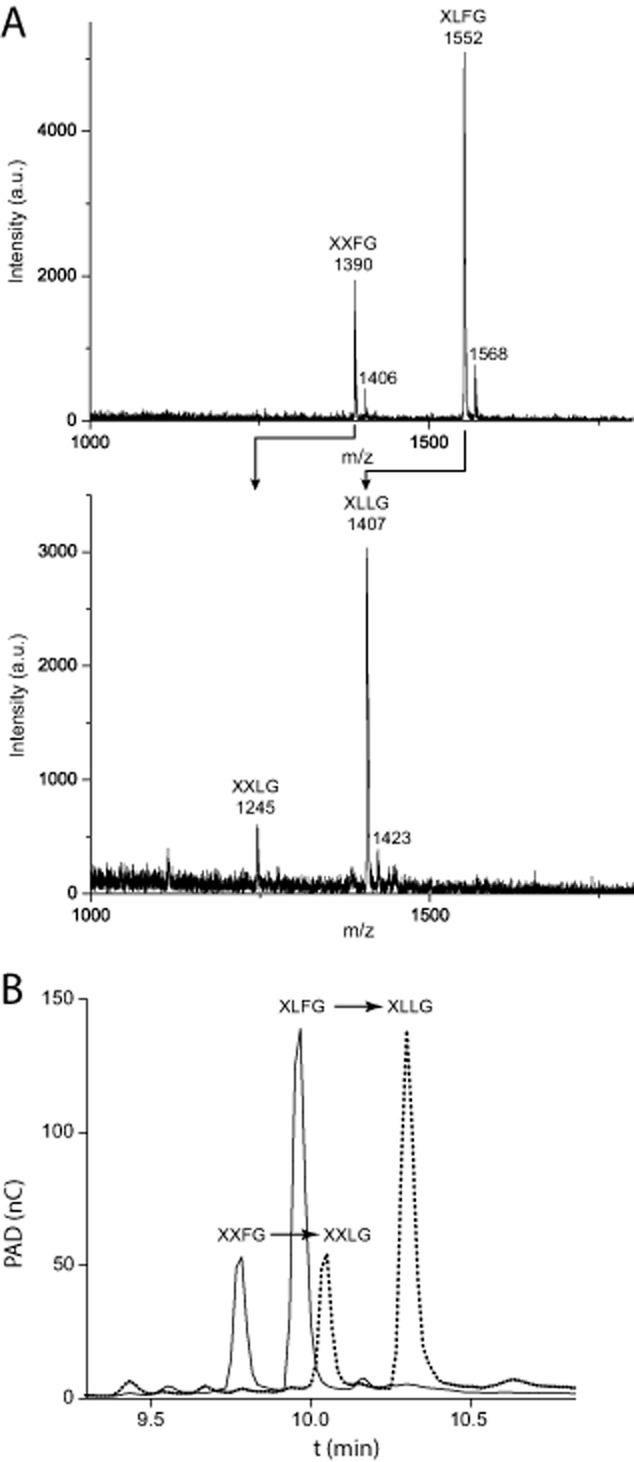
Analysis of products from *Cj*Afc95A activity on fucosylated xyloglucan oligosaccharides. A. MALDI-TOF spectra of the hydrolysis of a preparation containing XLFG and XXFG into XLLG and XXLG, respectively, after incubation with *Cj*Afc95A. B. HPAEC-PAD chromatograms of the same conversion. Observed *m/z* values are consistent with [M+Na]^+^ adducts; minor amounts of [M+K]^+^ adducts are also observed.

*Cj*Afc95A did not cleave any of the four PNP glycosides tested (*vide supra*), but readily hydrolysed l-Fuc-α-CNP. Using this substrate, the pH-rate profile was classically bell-shaped with a pH optimum of pH 6.5 in citrate buffer (Supplemental Fig. S3). The observed *k_cat_*/*K_m_* value for *Cj*Afc95A (12.9 mM^−1^ s^−1^; Table 1 and Fig. S4) compares favourably with that of a *Bifidobacterium longum* subsp. *infantis* GH95 member (0.12 mM^−1^ s^−1^) (Sela *et al.*, 2012). On natural substrates, product analysis by HPAEC-PAD and MALDI-TOF (Fig. 3) indicated that XLFG and XXFG were converted to XLLG and XXLG respectively. This specificity is similar to characterized GH95 α-l-fucosidases from the plants *A. thaliana* and *L. longiflorum*, both of which are active on fucosylated XyGOs (Ishimizu *et al.*, 2007; Leonard *et al.*, 2008).

The release of fucose from both the polysaccharide and the mixture of fucosylated XyGOs was also quantified by HPAEC-PAD (Table 1). Although the kinetics are confounded by the two-component XyGO mixture, the relative apparent *k_cat_/K_m_* value for the oligosaccharides was sevenfold higher than that for l-Fuc-α-CNP, due to a 10-fold lower apparent *K_m_* value. This suggests that carbohydrate-binding interactions in the positive enzyme subsites significantly enhance catalysis (for subsite nomenclature see Davies *et al.*, 1997). Indeed, the intrinsic leaving-group ability of 2-chloro-4-nitrophenol [p*K*_a_ 5.45 (Ibatullin *et al.*, 2008)] is potentially 10 orders of magnitude greater than a sugar hydroxyl group [p*K*_a_ *c*. 16 (Damude *et al.*, 1996)]. Similar strong effects of extended XyGO binding in positive enzyme subsites are exhibited by the α-xylosidase in the locus (Larsbrink *et al.*, 2011; Silipo *et al.*, 2012).

### The tertiary structure of *Cj*Bgl35A reveals the molecular basis for substrate recognition

To complement our structure–function analysis of *Cj*Xyl31A (Larsbrink *et al.*, 2011; Silipo *et al.*, 2012), we wished to complete the tertiary structural characterization of the remaining GHs encoded by the putative *C. japonicus* XyGUL to reveal the determinants of xyloglucan recognition. Whereas *Cj*Bgl35A has proven amenable to crystallography, *Cj*Afc95A has thus far resisted our attempts, despite extensive effort.

**Figure 4:**
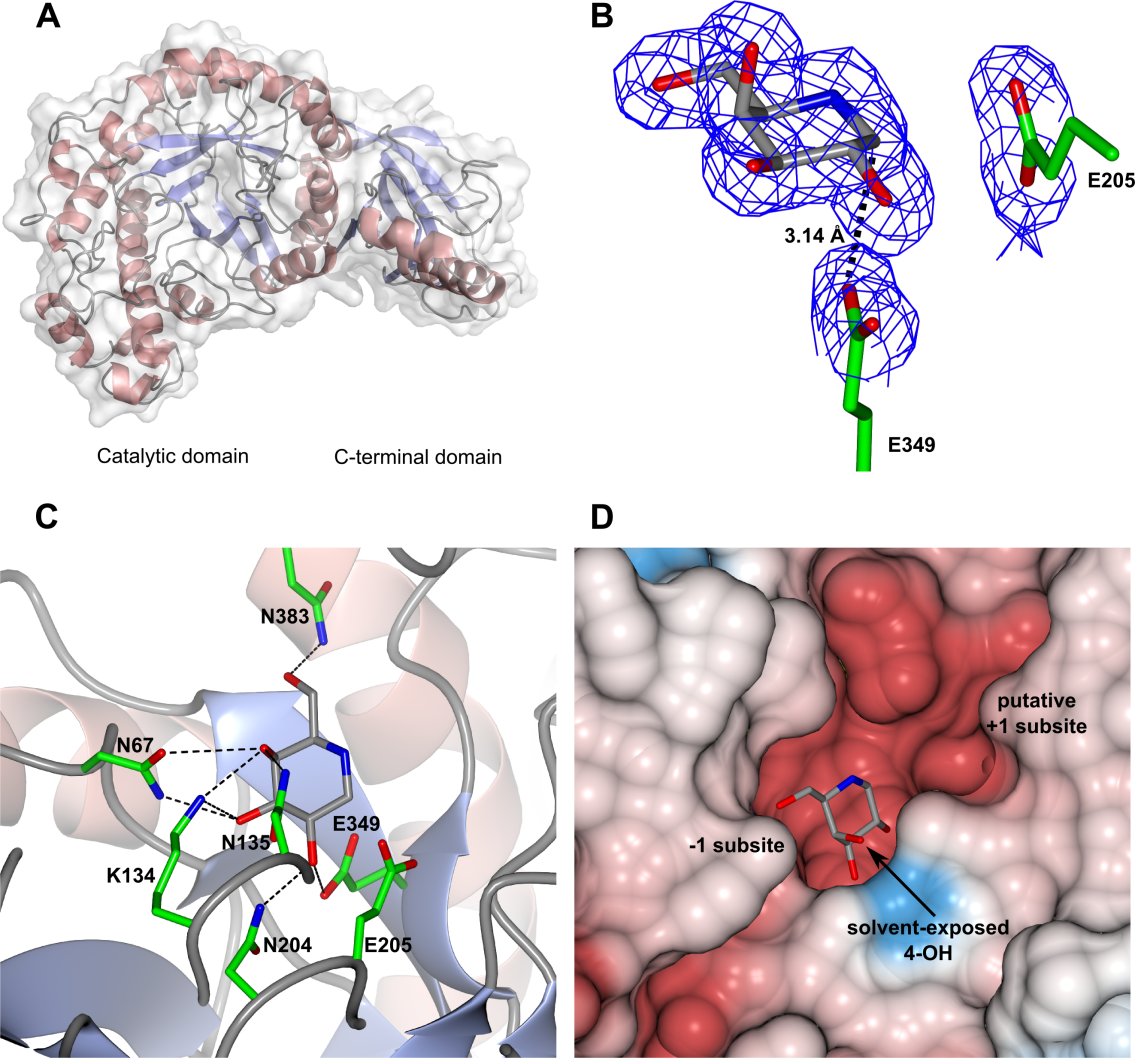
Crystallography of *Cj*Bgl35A. A. The tertiary structure viewed along the barrel axis of the N-terminal catalytic domain. The mixed α/β motif of the C-terminal domain is indicated, while a transparent surface overlay illustrates the rigid nature of the domain interface. B. 1-Deoxygalactonojirimycin (DGJ) bound in the active site (carbon atoms in grey); the proposed catalytic residues, E349 and E205, are also illustrated (carbon atoms in green). The pseudoanomeric position of the ligand can be observed approximately 3.14 Å from the carboxylate group of E349, the likely catalytic nucleophile. Electron density is depicted as a maximum-likelihood weighted 2F_o_−F_c_ synthesis contoured at 0.29 e Å^−3^ (1.0 σ). C. Additional residues in the −1 subsite interacting with GDJ. D. Surface representation, coloured according to electrostatic potential, showing the position of DGJ within the −1 subsite. A further cavity immediately adjacent to the pseudoanomeric position of DGJ is suggested to be the +1 subsite, accommodating the branched oligosaccharide substrates within the active-site cleft. Panel A was assembled using PyMOL version 1.3r1 (Schrödinger, LLC), while panels B–D were created using CCP4Mg (McNicholas *et al.*, 2011).

The three-dimensional structure of *Cj*Bgl35A reveals a relatively unusual two-domain architecture comprising an N-terminal catalytic (β/α)_8_ (TIM) barrel domain (residues 37–419), appended to a smaller, C-terminal, mixed α/β structure (residues 420–575) consisting of two short α helices and nine β strands (Fig. 4 and Supplemental Table S2; PDB ID 4D1I). Given its poor sequence similarity to other GH35 enzymes (*vide supra*), this two-domain fold exhibits low homology to other currently known protein structures; only one significant structural match to another bacterial GH35 β-galactosidase, from *Caulobacter crescentus* strain CB15, was found. Analysis using the Dali server (Holm and Rosenström, 2010) shows Bgl35A and the *C. crescentus* enzyme (PDB accession code 3U7V) to have a Z-score of 61.5, an r.m.s.d. value of 1.2 Å mapped across 508 Cα positions, and a sequence identity of 56%. The next most-similar protein (GH5 endo-β-mannanase, PDB code 1QNO) has a Z-score of 25.1, an r.m.s.d. value of 3.0 Å across 289 Cα positions, and a sequence identity of just 12%.

The (β/α)_8_ structure of the *Cj*Bgl35A N-terminal catalytic domain reveals a centrally positioned cleft running laterally across the open end of the barrel. This topology appears to be consistent with the requirement for binding of extended, branched oligo/polysaccharide substrates such as XyGOs and/or XyG and thus the cleft was anticipated to contain the catalytic active site. Subsequent soaking of native Bgl35A crystals with the iminosugar 1-deoxygalactonojirimycin [DGJ; 1,5-Dideoxy-1,5-imino-d-galactitol (Paulsen *et al.*, 1980)] confirmed both the location of the active centre and, importantly, unveiled crucial ligand-protein interactions including the identity of the likely catalytic amino acids (Fig. 4B and C, and Supplemental Table S2; PDB ID 4D1J). The *K*_d_ for this interaction in solution was determined to be 485 ± 42 nM by ITC (Fig. S5).

In the complex structure, DGJ is located within a deep cavity, approximately at the centre of the proposed active-site cleft. DGJ is co-ordinated in this −1 subsite by a complex hydrogen-bonding network mediated by the side-chain groups of residues N67 (interacting with O3), K134 (O3 and O4), N135 (O4), N204 (O2), and N383 (O6) (Fig. 4C). The side-chains of E205 and E349 occupy classical positions expected for the catalytic pair, comprising a proton donor-acceptor and nucleophile consistent with the retaining mechanism expected for all GH35 members (Fig. 4B). Furthermore, the position of the catalytic proton donor-acceptor residue is consistent with an *anti*-protonation trajectory (Heightman and Vasella, 1999).

A pocket positioned directly adjacent to the pseudoanomeric position of DGJ is proposed to be the +1 catalytic subsite that is responsible for binding side-chain xylosyl moieties present on XyG/XyGOs. Curiously, the apparent solvent-exposed nature of the non-reducing end of the ligand suggests the possibility to accommodate extended substrates, since the axial 4-OH position of the six-membered ring points directly out into the large cleft (Fig. 4D). However, in fucosylated XyGOs such as XXFG and XLFG, the pendant fucosyl residue is α(1→2)-linked to the galactosyl residue (Fig. 1). Steric considerations (Fig. 4D) therefore explain the requirement for *Cj*Afc95A to first remove the terminal Fuc from such XyGOs before the underlying Gal can be addressed by *Cj*Bgl35A (Fig. 2).

Despite the observation of separate protein domains (Fig. 4A), it seems likely that these together solely constitute a catalytic module, i.e. a distinct biochemical/biological function is not indicated. Structure/sequence homology searching of the C-terminal region in isolation produced no significant results that would indicate similarity to known regulatory or carbohydrate-binding modules (CBMs), despite the observation of a trough-like surface somewhat reminiscent of a substrate recognition cleft (Gilbert *et al.*, 2013). In addition, the observation that the early part of the C-terminal domain is formed from a distortion of the final helix of the barrel domain, and that the initial β-strand of this domain stacks directly against the penultimate barrel helix, strongly suggests a rigid, monolithic structure.

### Genetic analysis defines the biological importance of the XyGUL

With a firm understanding of the determinants of substrate recognition by the glycoside hydrolases *Cj*Xyl31A (Larsbrink *et al.*, 2011), *Cj*Bgl35A, and *Cj*Afc95A, we sought to confirm that the putative XyGUL indeed constituted a co-ordinately regulated locus. Further, we were interested to determine whether this multi-gene locus was exclusively responsible for XyG saccharification by *C. japonicus*.

### Transcriptional analysis

**Figure 5:**
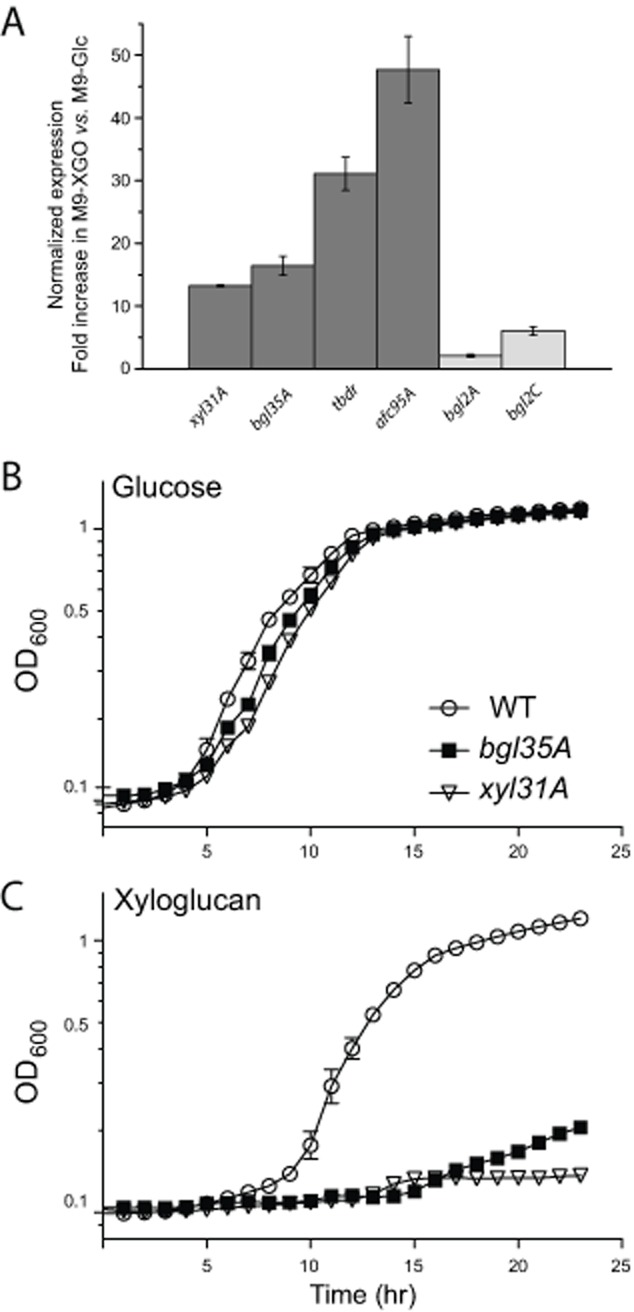
Transcriptomic and reverse genetic analysis of the *C. japonicus* xyloglucan utilization locus (XyGUL). A. Normalized increase in expression of the locus genes (cf. Fig. 1) in cells grown on M9-XyGO medium versus to M9-Glc medium, compared with other predicted galactosidase-encoding (*bgl2A,* *bgl2B,* *bgl2C*) and fucosidase-encoding (*afc95B*) genes (Deboy *et al.*, 2008). *bgl2B* and *afc95B* had identical expression in both growth conditions and therefore served as reference genes. Error bars indicate the standard error of the mean. B and C. Growth analysis of *C. japonicus* wild-type (WT) and *bgl35A* and *xyl31A* knockout mutant strains in MOPS minimal medium with either 0.25% glucose (B) or 0.25% xyloglucan (C) as sole carbon sources. All growth experiments were performed in biological triplicate; error bars represent the standard deviation of the mean (in many cases the error bars are smaller than the data point marker).

Quantitative PCR (qPCR) was performed on all four predicted XyGUL genes (Fig. 1; *xyl31A, bgl35A, tbdr, afc95A*) to monitor possible upregulation during growth on minimal medium containing tamarind XyGOs versus a glucose control. Additional predicted β-galactosidases and α-l-fucosidases found in the *C. japonicus* genome (Deboy *et al.*, 2008) (*bgl2A, bgl2B, bgl2C* and *afc95B*), but not in the putative XyGUL, were also included to determine their potential involvement in XyG deconstruction. Of these, the predicted β-galactosidase gene, *bgl2B*, and the other predicted α-l-fucosidase gene, *afc95B*, showed the same level of expression in both growth conditions, and were therefore used as reference genes. The four XyGUL genes were all clearly upregulated 12- to 45-fold when *C. japonicus* was grown in M9-XyGO medium (Fig. 5A).

The predicted β-galactosidase genes *bgl2A* and *bgl2C*, which are not in the putative XyGUL were also slightly (< 5-fold) upregulated in the M9-XyGO cultures (Fig. 5A). However, genome analysis suggests that these genes are unlikely to be purposely involved in XyG catabolism and may instead be responding to a general sensing signal due to galactose released by the XyGUL enzymes. For example, *bgl2A* (CJA_0496) is located together with a predicted *endo*-1,4-galactanase, *gal53A-2* (CJA_0497) and a TonB-dependent receptor (CJA_0498), and might instead be primarily involved in (arabino)galactan metabolism. *bgl2C* (CJA_2610) is co-located with a predicted chitinase, chi18D (CJA_2611), which may point towards a principal function related to fungal cell wall or insect carapace degradation.

To assay whether any of the locus genes were transcribed as a polycistronic mRNA, PCR was performed in an attempt to amplify the regions between each of the genes in the cDNA, using the primers from the qPCR study. The *xyl31A* and *bgl35A* genes were found to be co-transcribed on one bicistronic mRNA strand (data not shown). This observation is supported by the similar expression levels of these two genes indicated by qPCR (Fig. 5A) and the distance between the genes (41 bases), which is unlikely to accommodate a promoter region. Similar analyses of the *bgl35A/tbdr* and *tbdr/afc95A* pairs suggested that the TonB-dependent receptor gene and *afc95A* are transcribed individually. The non-coding regions directly upstream of these genes are 88 and 199 bases in length respectively, and are therefore large enough to contain individual promoters.

### Reverse genetics of *xyl31A* and *bgl35A*

To assess physiological function, *xyl31A* and *bgl35A* gene-disruption mutants were generated, confirmed by PCR (Fig. S6), and assayed for growth on tamarind xyloglucan. Inactivation of *xyl31A* mutation results in a severe growth defect, with a growth rate of 0.06 h^−1^ (peak OD_600_ = 0.13) on xyloglucan, compared to 0.41 h^−1^ (peak OD_600_ = 1.2) of the wild-type strain (Fig. 5B and C). As expected from its bicistronic relationship with *xyl31A*, disruption of *bgl35A* likewise results in a severe growth defect (growth rate 0.07 h^−1^, peak OD_600_ = 0.21). Both mutants grow in a wild-type manner on the polysaccharides xylan (from beechwood), carboxymethyl cellulose, and pectin (from apple).

### Subcellular localization of XyGUL function

We previously demonstrated that *Cj*Xyl31A is cell membrane-bound, with attachment likely mediated through N-terminal lipidation (Larsbrink *et al.*, 2011). In contrast, LipoP (Juncker *et al.*, 2003) predicts that neither *Cj*Bgl35A nor *Cj*Afc95A are N-terminally lipidated. Furthermore, SignalP (Petersen *et al.*, 2011) fails to identify a signal peptide in *Cj*Afc95A. To further refine our spatial model of XyG utilization in *C. japonicus*, the subcellular locations of *Cj*Bgl35A, and *Cj*Afc95A were investigated by specific chromogenic assays of cell fractions. Notably, no significant α-l-fucosidase activity could be detected in any of the protein fractions from the M9-Glc cultures (Table 2). Likewise, β-galactosidase activity was absent in the secreted protein fraction from M9-Glc cultures, while only very weak activity was observed in the periplasmic and cytoplasmic protein fractions. In contrast, growth on M9 medium containing XyGOs to induce XyGUL expression resulted in a significant increase in both activities in all three fractions. The specific activity levels were however clearly highest in the periplasmic fractions versus the secreted and cytoplasmic fractions (activity in these fractions, which is at least eightfold lower in all cases, may represent some degree of cross-contamination).

**Table 2:** Specific galactosidase and fucosidase activities for proteins produced under different growth conditions

Growth medium	Substrate	Specific activity in cell fractions (μM released product·s^−1^·μg protein^−1^)		
		Secreted	Periplasmic	Intracellular
M9-XyGO	Gal-β-PNP	1.25 ± 0.14	18.3 ± 3	1.0 ± 0.3
	l-Fuc-α-CNP	160 ± 47	1400 ± 160	28.8 ± 6.6
M9-Glc	Gal-β-PNP	n/d	0.86 ± 0.05	0.058 ± 0.003
	l-Fuc-α-CNP	n/d	n/d	n/d

## Discussion

**Figure 6:**
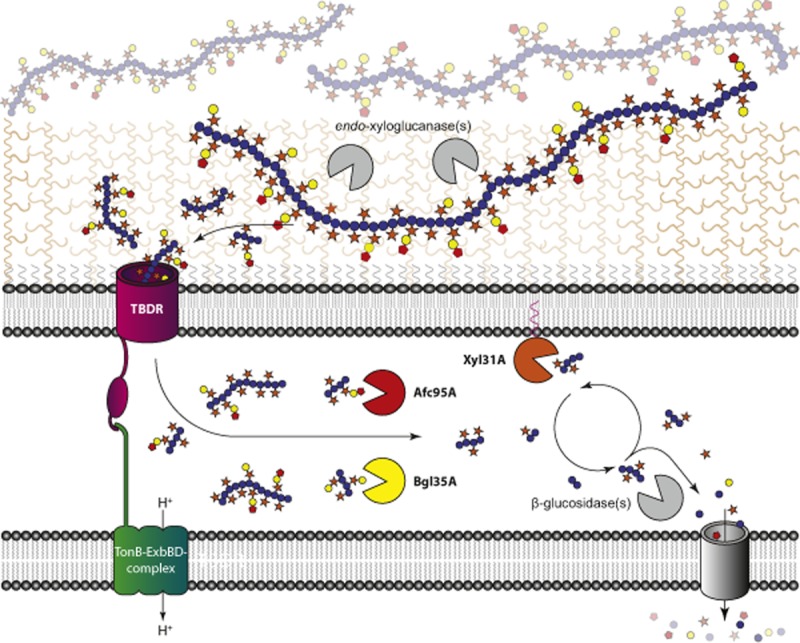
Proposed pathway of (fucogalacto)xyloglucan degradation by *C. japonicus*. Sugar symbols are as follows: Glc – blue circles, Xyl – orange stars, Gal – yellow circles, Fuc – red pentagons. Secreted enzymes with *endo*-xyloglucanase activity depolymerize the polysaccharides into xyloglucan oligosaccharides which are imported into the periplasm by the TonB-dependent receptor of the locus. In the periplasm, *Cj*Bgl35A and *Cj*Afc95A strip off galactose and fucose from the oligosaccharides respectively. In concert, *Cj*Xyl31A and an unknown β-glucosidase cleave off terminal xylose and glucose residues from the non-reducing end in an iterative manner.

Transcript and reverse genetics analyses allow us to conclude that *xyl31A, bgl35A, tbdr*, and *afc95A* (Fig. 1) constitute a xyloglucan utilization locus (XyGUL), which is the primary genetic determinant conferring *C. japonicus* with the ability to saccharify this ubiquitous plant cell wall polysaccharide. Together with substrate specificity and structural analysis of the encoded glycoside hydrolases, subcellular localization data allow us to propose an updated model of XyG degradation in *C. japonicus* (Fig. 6). In this model, XyG polysaccharide is hydrolysed into component oligosaccharides by one or more *endo*-xyloglucanase(s). The liberated XyGOs are then imported into the periplasm via the TBDR (Ferguson and Deisenhofer, 2002; Koebnik, 2005; Dejean *et al.*, 2013), where the *exo*-glycosidases *Cj*Xyl31A, *Cj*Bgl35A, and *Cj*Afc95A work in concert, together with a currently unidentified β-glucosidase(s), to yield monosaccharides for further catabolism.

Notably, structural enzymology provides some of the strongest support for this model. The α-xylosidase, *Cj*Xyl31A, has a strict non-reducing-end specificity due to a deep, pocket-shaped active site and thus cannot access xylosyl residues positioned along the intact polysaccharide chain (Larsbrink *et al.*, 2011). Likewise, the apparent *k_cat_/K_m_* value of *Cj*Bgl35A for the oligosaccharide XLLG is three orders of magnitude higher than for tamarind XyG polysaccharide (Table 1), which, together with the observed pocket topology of the active-site (Fig. 4), suggests that cleavage of the polysaccharide to XyGOs also occurs prior to the action of this enzyme. As highlighted above, steric limitations of the *Cj*Bgl35A active-site also indicate that *Cj*Afc95A must remove terminal fucosyl residues prior to fully enable degalactosylation by *Cj*Bgl35A.

It is particularly interesting to note the obvious differences between the XyGUL system of *C. japonicus vis-à-vis* the polysaccharide utilization loci (PULs) of Bacteroidetes (Koropatkin *et al.*, 2012). A recently characterized XyGUL from *B. ovatus* comprises a complete cohort of GHs sufficient to address each linkage in solanaceous (arabinogalacto)xyloglucan, including both a keystone *endo*-xyloglucanase and six diverse *exo*-glycosidases, in addition to a TBDR, a hybrid two-component sensor/regulator, and two cell-surface carbohydrate-binding proteins (Larsbrink *et al.*, 2014; Terrapon and Henrissat, 2014). In contrast, the *C. japonicus* XyGUL – in addition to being alternatively directed towards dicot (fucogalacto)xyloglucan – is significantly less complete: It does not encode the requisite *endo*-xyloglucanase(s) necessary to initiate the saccharification pathway by polysaccharide backbone cleavage, yet *C. japonicus* does secrete significant *endo*-xyloglucanase activity into the medium under XyGO-induction (Larsbrink *et al.*, 2011). Likewise, the *C. japonicus* XyGUL does not encode a β-glucosidase, which would be required to digest the β(1→4)-glucan backbone of the XyGOs. Finally, the XyGUL appears to lack a substrate sensor/transcriptional regulator analogous to that which regulates xylan-specific genes in *C. japonicus* (Emami *et al.*, 2009). Thus, although the *C. japonicus* XyGUL shares a theme of genetic colocalization and co-regulation with the Bacteroidetes PULs, it is notably less evolved with respect to complexity and completeness. It is also notable that the TBDRs of the *C. japonicus* and *B. ovatus* XyGULs do not share any significant sequence similarity, despite predicted functional homology based on their association with a similar complement of GHs.

In general, the colocalization and co-ordinated regulation of CAZyme-encoding genes in *C. japonicus* has not been widely studied. Indeed, annotation of the *C. japonicus* genome revealed that many of these genes are not clustered on the chromosome, although interesting exceptions can be observed (Deboy *et al.*, 2008). For example, early work by Gilbert, Hazlewood and co-workers revealed that *endo*-xylanase B of GH10 (CJA_3280) and α-l-arabinofuranosidase C of GH62 (CJA_3281) are encoded by adjacent genes in *C. japonicus*. Post-genomic re-examination by McClendon *et al.* has revealed that these are likely to constitute part of a Xylan Utilization Locus that additionally encodes a predicted *endo*-xylanase of GH30 (CJA_3279), feruloyl esterase D (Fee1B) of CE1 (CJA_3282) (Ferreira *et al.*, 1993), a predicted β-galactosidase of GH98 (CJA_3286), and a second predicted feruloyl esterase (Fee1A) of CE1 (CJA_3287) (McClendon *et al.*, 2011). Likewise, the *endo*-xylanase Xyn10D (CJA_2888) (Emami *et al.*, 2009) is located between the α-glucuronidase GlcA67A (CJA_2887) (Nagy *et al.*, 2003) and the acetylxylan esterase Axe2B (CJA_2889) (Zhang *et al.*, 2014). On the other hand, the reason for colocalization of other genes is sometimes less obvious: the broad-specificity xylan-active α-l-arabinofuranosidase Abf51A (CJA_2769) (Beylot *et al.*, 2001) is found in tandem with the *endo*-mannanase Man26A (CJA_2770) (Hogg *et al.*, 2001), which perhaps suggests a greater diversity of mannan substructures than previously appreciated. Regardless, we posit that increased consideration of CAZyme colocalization within genomes can significantly facilitate understanding the basic biology of *C. japonicus* and other bacteria, as well as advance the discovery of novel enzyme cohorts for biotechnological applications (Martens *et al.*, 2014). For example, a recent study of a mannan-degrading locus of *Bacteroides fragilis* has identified a syntenic locus in *C. japonicus* (CJA_0241–0246) putatively comprising a sugar/cation symporter, a mannosyl-glucose phosphorylase, a mannobiose 2-epimerase, a GH5 *endo*-mannanase and a GH27 α-galactosidase (Deboy *et al.*, 2008; Senoura *et al.*, 2011).

The data presented here shed new light on the genetic, biochemical, and structural basis of hemicellulose utilization by *C. japonicus* and, moreover, reveal a novel, matched set of glycosidases for biotechnological applications. Indeed, the importance of xyloglucan saccharification has been arguably under-appreciated in the context of biofuel production (Gilbert *et al.*, 2008), although recent studies are beginning to address this issue (Hu *et al.*, 2013; Jabbour *et al.*, 2013), buoyed by an increased understanding of specific, xyloglucan-active enzymes (Gilbert *et al.*, 2008; Ariza *et al.*, 2011; Larsbrink *et al.*, 2011; Eklöf *et al.*, 2012). Interestingly, the xyloglucan utilization locus described here encodes neither the *endo*-xyloglucanase(s) nor the β-glucosidase(s) required for full degradation of XyG. The proteins necessary for sensing XyG in the environment are also presently unknown. Future studies from our collaboration will focus on the identification of the corresponding genes elsewhere in the genome, in addition to functional studies on the TonB-dependent receptor of the locus.

## Experimental procedures

Ultrapure water, purified on a Milli-Q system (Millipore) to a resistivity of ρ > 18.2 MΩ cm, was used in all experiments. Galactose, lactose, Gal-β-PNP, Glc-β-PNP, Xyl-β-PNP and l-Ara*f*-α-PNP were purchased from Sigma. l-Fuc-α-CNP and fucose were purchased from Carbosynth. Tamarind xyloglucan (XyG) was purchased from Megazyme. XLLG was prepared from tamarind XyG as described previously (Greffe *et al.*, 2005). Lettuce XyG and XLFG were isolated as described below. *C. japonicus* Ueda107 was obtained from the National Collections of Industrial, Marine, and Food Bacteria (Aberdeen, Scotland).

### Cloning of *Cj*Bgl35A and *Cj*Afc95A

The open reading frames encoding *Cj*Bgl35A and *Cj*Afc95A (GenBank Accession No. ACE85180.1 and ACE83895.1 respectively) were amplified by PCR from genomic DNA of *C. japonicus* Ueda107 using Phusion polymerase (Finnzymes), using forward primers incorporating a CACC overhang to enable TOPO cloning, and reverse primers excluding stop codons (Eurofins MWG Operon; Supplemental Table S1). The forward primer for *Cj*Bgl35A was designed to exclude the predicted native signal peptide (residues 1–36), while no signal peptide was predicted for *Cj*Afc95A (Petersen *et al.*, 2011). The PCR products were cloned into the pENTR/SD/D-TOPO entry vectors (Invitrogen) and recombined into pET-DEST42 destination vectors (Invitrogen) following the manufacturer's instructions.

Initial protein crystals produced from the pET-DEST42 construct, featuring a larger C-terminal tag, yielded only relatively weak diffraction to approximately 4.0 Å (data not shown) and proved difficult to optimize further. As such, primers were designed to allow recloning of the gene fragment encoding *Cj*Bgl35A into a pET28a vector modified for Ligation Independent Cloning (LIC), and featuring a shorter, N-terminal hexahistidine tag (Eurofins MWG Operon; Supplemental Table S1). The PCR product was cloned into a pre-prepared linear vector stock using the InFusion-HD cloning kit (Clontech) according to the manufacturer's instructions.

### Recombinant gene expression and protein purification

pET-DEST42 constructs were transformed into *E. coli* BL21(DE3) by electroporation, and proteins were produced and purified by immobilized metal affinity chromatography (IMAC) following an established protocol (Larsbrink *et al.*, 2011). The modified pET28a-Bgl35A construct was transformed into *E. coli* TUNER(DE3) cells via the heat-shock method, with recombinant protein purified by IMAC and size-exclusion chromatography. Protein purity was verified by SDS-PAGE. Protein concentration was determined from *A*_280_ values; the extinction coefficients used for *Cj*Bgl35A and *Cj*Afc95A were 118260 and 151845 M^−1^ cm^−1^ respectively [ProtParam server (Gasteiger *et al.*, 2005)].

### Crystallization, data collection and structure solution of *Cj*Bgl35A

Crystals of *Cj*Bgl35A suitable for full X-ray data collection were grown using hanging drop vapour diffusion at 19°C, with equal volumes of pure protein and reservoir solution (2.6 M sodium acetate pH 7.2). Ligand complex formation with GDJ was achieved by soaking native crystals in 10 mM GDJ (final) for a period of approximately 1 h. Since concentrated sodium acetate present within the mother-liquor solution proved an adequate cryo-protectant, no additional solvents were added to crystals prior to flash cooling in liquid N_2_. Full diffraction data for both native and ligand complex *Cj*Bgl35A crystals were collected at beamline I03 of the Diamond Light Source (Didcot, Oxfordshire, UK). Measured reflection intensities were indexed, integrated and scaled using XDS (Kabsch, 2010a, b) and the CCP4 suite (Winn *et al.*, 2011) implementation of Aimless. The structure of *Cj*Bgl35A was solved by molecular replacement with PHASER (McCoy *et al.*, 2007), employing the co-ordinates of the *C. crescentus* GH35 (PDB code 3U7V) as a phasing model. An initial atomic model was constructed using the CCP4 (Winn *et al.*, 2011) implementation of Buccaneer and refined via the maximum-likelihood method using numerous cycles of REFMAC (Murshudov *et al.*, 2011; Winn *et al.*, 2011), with additional manual correction using COOT (Emsley *et al.*, 2010). The structure of *Cj*Bgl35A in complex with GDJ was produced by refining the scaled data (processed as above) against the finalized model of the native protein and visually inspecting calculated electron density maps for evidence of ligand binding. The final atomic model of the complex was subsequently refined and corrected as above. Models and structure factors for both native *Cj*Bgl35A and the GDJ complex have been deposited into the PDB with respective accession codes 4D1I and 4D1J.

### High-performance anion-exchange chromatography with pulsed amperometric detection (HPAEC-PAD)

Oligo- and monosaccharides were analysed on a Dionex ICS-3000 HPLC system operated by Chromelion software version 7 (Dionex) using a Dionex CarboPac PA200 column for Gradient A and a Dionex CarboPac PA1 column for Gradient B. Solvent A was water, solvent B was 1 M sodium hydroxide and solvent C was 1 M sodium acetate. The gradients used were as follows: Gradient A: 0 to 5 min, 10% B, 2% C; 5 to 12 min, 10% B and a linear gradient from 2% to 30% C; 12 to 12.1 min, 50% B, 50% C; 12.1 to 13 min, an exponential gradient of B and C back to initial conditions; 13 to 17 min, initial conditions. Gradient B: column pre-conditioned prior to injection by −13 to −3 min, 12% B, 6.8% C; −3 to 0 min, 100% A; 0 to 25 min, 100% A.

### Matrix-assisted laser desorption/ionization-time-of-flight (MALDI-TOF) analysis of oligosaccharides

MALDI-TOF was performed on oligosaccharide samples using a Voyager-DE STR instrument (Applied Biosystems) in positive linear mode with an acceleration of 20 kV and an extraction delay time of 150 ns. The samples were prepared by mixing equal amount of sample (1 μl) with the matrix (10 mg ml^−1^ 2,5 dihydroxybenzoic acid in 1:1 acetonitrile : H_2_O, containing 0.05% trifluoroacetic acid).

### Xyloglucan extraction from iceberg lettuce

Using a modified and scaled-up version of a published protocol (Hsieh and Harris, 2009), XyG polysaccharide was extracted from approximately 500 g of fresh iceberg lettuce leaves, obtained from a local grocery store. The plant material was homogenized in 70% aqueous ethanol using a high-speed blender, collected by filtration using Miracloth (Millipore) and ground to a fine powder in liquid nitrogen using a ceramic mortar and pestle. Alcohol-soluble polysaccharides and smaller sugars were removed by repeated 70% ethanol wash and filtration steps. Non-cellulosic polysaccharides were extracted in 6 M sodium hydroxide, containing 1% sodium borohydride to prevent alkaline peeling, followed by neutralization with acetic acid.

Ethanol was added to the neutralized solution to a final concentration of 70% to precipitate polysaccharides, followed by centrifugation at 24 000 *g* for 15 min. The supernatant was discarded and the pellet washed three times with 70% ethanol. The washed pellet was dissolved in water and loaded onto a Q Sepharose column (GE Lifesciences), pre-equilibrated with 10 mM imidazole (pH 7.0) to bind charged polysaccharides (e.g. pectins) to the matrix. Neutral hemicellulosic polysaccharides were eluted in three column volumes of the same buffer. The resulting hemicellulose-containing fraction was incubated with 150 units of the xylanase *Cj*CBM22-GH10 (Xyn10A) and 150 units of mannanase 26A (both purchased from NZYtech) for 16 h at 37°C, to hydrolyse contaminating xylan and mannan respectively. The resulting oligosaccharides were subsequently removed in the supernatant following ethanol precipitation of the lettuce XyG (final yield 180 mg after lyophilization from water). Prior to analysis by HPAEC-PAD (gradient B), 1 mg of lettuce XyG was hydrolysed by incubation with 2 M trifluoroacetic acid (TFA) for 3 h at 120°C (1 ml total volume). Hydrolysis products were vacuum-dried and re-suspended in deionized water, followed by filtration (0.2 μm). The lettuce xyloglucan comprised Glc, Xyl, Gal and Fuc, with pectin derived contaminations of approximately 5%.

In a second extraction, fucosylated xylogluco-oligosaccharides (XyGOs) were obtained via a similar procedure lacking the ion exchange step. Seven hundred and fifty milligrams of lyophilized crude lettuce XyG was dissolved in 100 ml 50 mM ammonium acetate, pH 5.5, to which was added 1.28 mg *B. ovatus Bo*GH5A *endo*-xyloglucanase (Larsbrink *et al.*, 2014). The reaction was incubated overnight at 35°C followed by lyophilization (dry weight 0.3 g). XyGOs were dissolved in 1 ml water and loaded onto a 100 cm XK16 column packed with 200 ml Bio-Gel P-2 Gel (Bio-Rad), which had been equilibrated with water. Eluted fractions were analysed by MALDI-TOF and fractions containing predominantly XXXG, XXFG and XLFG, respectively, were pooled and lyophilized.

### Enzyme assays

All assays were carried out at 25°C, at the pH optimum of the respective enzyme. Curve fitting and processing of kinetic data were performed using Origin 8 software (OriginLab).

### pH dependence

Measurements of the pH-dependence of *Cj*Bgl35A and *Cj*Afc95A were performed with Gal-β-PNP and l-Fuc-α-CNP as substrates, respectively, using the assays described below. Buffers (50 mM) are indicated in Figs S1 and S3.

### Chromogenic assays

Activities on PNP glycosides were analysed by a stopped assay as previously described (Larsbrink *et al.*, 2011), using enzyme concentrations in the μM range for initial screens and several hours incubation. For initial rate kinetics on Gal-β-PNP for *Cj*Bgl35A, 42 nM enzyme was used and reactions were stopped after 10 min. Assays utilizing l-Fuc-α-CNP were monitored continuously for the release of 2-chloro-4-nitrophenolate using a Cary 300 spectrophotometer (Agilent Technologies). For the determination of the kinetic parameters of *Cj*Afc95A, 22 nM enzyme was used. An extinction coefficient of 12 936 M^−1^ cm^−1^, determined from a standard curve, was used to calculate product concentration from *A*_405_ values.

### HPAEC-PAD-based assays

Enzymatic reactions on XyG polysaccharides and XyGOs were performed in 50 μl reactions, containing 50 mM buffer at the pH optimum of the assayed enzyme, and were stopped by addition of 2 μl of 5 M sodium hydroxide prior to HPAEC-PAD analysis. For the reaction of *Cj*Bgl35A on XLLG, 2.7 nM enzyme was used, the reaction was terminated after 10 min. For the reaction of *Cj*Bgl35A on tamarind XyG, 0.16 μM enzyme was used, the reaction was terminated after 130 min. For the reaction of *Cj*Afc95A on XLFG, 0.64 nM enzyme was used, and the reaction was terminated after 15 min. For the reaction of *Cj*Afc95A on lettuce XyG, 270 nM enzyme was used, and the reaction was terminated after 20 min. To quantify the release of galactose or fucose from the reactions, the respective commercial monosaccharides were used as standards.

### Isothermal titration calorimetry (ITC)

Isothermal titration calorimetry (ITC) was performed with a MicroCal Auto-iTC200 system (GE Healthcare). Assays were conducted in triplicate at 25°C, with GDJ (390 μM) titrated into the ITC cell containing pure *Cj*Bgl35A (38.5 μM). Dissociation constants (*K*_d_) for each titration were subsequently calculated and averaged using the Origin 7 software package (MicroCal).

### Transcription analysis

Total RNA was extracted from cultures of *C. japonicus* grown in liquid M9 minimal media supplemented with either a mixture of tamarind XyGOs (4 g l^−1^) or glucose (10 g l^−1^). Five millilitres overnight cultures were used to inoculate 200 ml cultures of the same media, and the OD_600_ of the cultures were monitored. When the OD_600_ had reached 1, corresponding to a late exponential growth phase (Gardner and Keating, 2010), 2 ml culture was added to 4 ml RNAprotect Bacteria Reagent (Qiagen) and the cells were harvested by centrifugation at 4300 *g* for 5 min. Total RNA was extracted using the RNeasy mini kit (Qiagen), following the manufacturer's instructions, including in-solution DNase treatment and subsequent clean-up. The final yield of total RNA was on average 42 μg for XyGO cultures and 21 μg for Glc cultures. One microgram of each biological sample was reverse transcribed into cDNA by the iScript cDNA Synthesis Kit (Bio-Rad). qPCR was performed on a CFX96 Touch instrument using Hard-Shell white well plates (Bio-Rad). Primers for the locus genes (Eurofins MWG Operon; Table S1), as well as the predicted β-galactosidase encoding genes *Cj*Bgl2A, *Cj*Bgl2B, *Cj*Bgl2C and the predicted α-l-fucosidase encoding gene *Cj*Afc95B were used to analyse the respective genes for each growth condition in biological duplicates and technical triplicates. The protocol used for amplification consisted of: 95°C 3 min, 40 cycles of 95°C, 15 s; 58°C, 15 s; 72°C, 15 s, followed by a melt curve (65°C to 95°C read in 0.5°C steps). The qPCR results were analysed using the Bio-Rad CFX Manager 2.0 software (Bio-Rad). To analyse whether the genes were transcribed as an operon in a single polycistronic mRNA strand, the primers from the qPCR study were used to amplify the regions between adjacent genes using *Phusion* polymerase (Finnzymes) and the constructed cDNA as template.

### Enzyme localization studies

The remaining cells from the minimal media cultures, above, were harvested by centrifugation at 4300 *g* for 10 min, and subjected to osmotic shock as described previously (Larsbrink *et al.*, 2011). The secreted proteins in the supernatant media were collected, concentrated and washed with 50 mM sodium phosphate, pH 7.0, using Amicon Ultra filter units (10 kDa cut-off; Millipore). The remaining cells were re-suspended in 5 ml sodium phosphate, pH 7.0, and lysed by sonication. The lysate was centrifuged at 25 000 *g* for 45 min and the resulting supernatant liquid was collected and concentrated with the same filter units and buffer as above to obtain soluble cytoplasmic proteins. The three fractions containing secreted, periplasmic, and cytoplasmic proteins were each assayed for β-galactosidase and α-l-fucosidase activities using the chromogenic assays described above; protein concentrations were measured by the Bradford method.

### Construction of targeted gene disruptions and strain growth analysis

Directed gene disruptions from integration of suicide plasmid pK18*mobsacB* (Schafer *et al.*, 1994) into the genome were facilitated by tri-parental mating as done previously (Gardner and Keating, 2010; 2012,). Confirmation of correct gene disruptions was performed by PCR using primers listed in Table S1. *C. japonicus* strains were grown on MOPS minimal medium (Neidhardt *et al.*, 1974) with glucose [0.25% (wt/vol)] or tamarind xyloglucan [0.25% (wt/vol)] as sole carbon sources. Growth assays were performed in a TECAN M200Pro at 30°C with vigorous shaking. Growth measurements were taken via optical density (OD) at 600 nm. Growth experiments were performed in biological triplicates.
