# Supplementary material for: A complex gene locus enables xyloglucan utilization in the model saprophyte Cellvibrio japonicus
Source: Mol Microbiol. 2014 Sep 17;94(2):418–33. doi: 10.1111/mmi.12776 (PMC4285296; doi:10.1111/mmi.12776)
Supplement: Supplementary file 1 — Supporting Information [file mmi0094-0418-SD1.pdf]

Supplementary Information

Table S1: Primers used for cloning, qPCR, and mutant strain confirmation

Primers for cloning	Sequence
Bgl35A_TOPO_F	CACCGCCGCACCACTTCCTGAG
Bgl35A_TOPO_R	CTGAACGCTATAGCTGGC
Afc955A_TOPO_F	CACCATGAGCGCAGAGGTG
Afc955A_TOPO_R	CTTCTGCATACTCAAGAGATACTC
Bgl35A_Lic-F	CATCACCACCACCACGCCGCACCACTTCCTGAGTTGTTATCC
Bgl35A_Lic-R	TGAGGAGAAGGCGCGTTACTGAACGCTATAGCTGGCCATTTTGACACG

Primers for qPCR	
Xyl31A_F	CCAAGGGTAATGTGCTGCTACAAG
Xyl31A_R	CCTGGCGCAACGCATAAGAATCG
Bgl35A_F	GGAACAAATTGAGCCAGTAGAGGG
Bgl35A_R	CCACAGCAACACCAACCTTACCTTG
TBDR_F	GCTCGTTTTGATGCGGAATATGATTTCG
TBDR_R	CCAGTGTTAATATTATCGGCTTCTTTCTCG
Afc95A_F	CAGCTGTTGGCACGCCATACG
Afc95A_R	CCAATAATGCTGGTGTTGCCAGTG
Bgl2A_F	CCAACAACTCCAGGTGGTACAGC
Bgl2A_R	GATTTTCACTATCGCTTCCGGCATTC
Bgl2B_F	CGCAATGTCTGGCTCACCAAAACC
Bgl2B_R	CATCCACCGCAATACGGGCAG
Bgl2C_F	GAATACCGTTTGCGTCGCTTGAAGG
Bgl2C_R	CCATGCGATCAGCCATATCCAGTAC
Afc95B_F	GCTGTGCCAGCATATCTGGTATCAC
Afc95B_R	GACAAAAAAGCGCGAGGCCTCG

Primers used for confirming the *bgl35A* and *xyl31A* strain knock-out mutations

*bgl35A*_P1	ATGCCACACCGATAATA
*bgl35A*_P2	CAATGACGTTCAAATTGA
*bgl35A*_P3	CAGGCGCTCGTAGAC
*bgl35A*_P4	ATGCCACACCGATAATA
*bgl35A*_P5	GTGTGGAATTGTGAGCG
*bgl35A*_P6	CAATGACGTTCAAATTGA
*bgl35A*_P7	GACAGTTTGATTTCTCCT
*bgl35A*_P8	TTAATAGCTTTACCCGC
*xyl31A*_P1	ACGGCTTATAAGGCA
*xyl31A*_P2	ATTTCGGTGGTAGCT
*xyl31A*_P3	CAGGCGCTCGTAGAC
*xyl31A*_P4	ACGGCTTATAAGGCA
*xyl31A*_P5	GTGTGGAATTGTGAGCG
*xyl31A*_P6	ATTTCGGTGGTAGCT
*xyl31A*_P7	TGAGCTTCCGTGATG
*xyl31A*_P8	GCATAACCGGAGTTATAC

Table S2: Data collection and refinement statistics for *Cj*Bgl35A native and 1-deoxygalactonojirimycin (DGJ) ligand complex structures.

	*Cj*Bgl35A (PDB 4D1I)	*Cj*Bgl35A-GDJ (PDB 4D1J)
**Data collection**		
Space group	P1	P1
Cell dimensions		
a, b, c (Å)	98.9, 115.8, 116.0	99.3, 116.1, 116.1
α, β, γ (°)	90.2, 90.3, 90.4	90.0, 90.1, 90.0
Resolution (Å)	46.0 – 1.80 (1.83 – 1.80)	46.0 – 1.80 (1.83 – 1.80)
R_{sym} or R_{merge}	0.063 (0.63)	0.051 (0.57)
$I/\sigma I$	8.7 (1.1)	9.8 (1.4)
Completeness (%)	96.4 (95.1)	97.1 (95.8)
Redundancy	1.8 (1.8)	2.2 (2.1)
**Refinement**		
Resolution (Å)	1.80	1.80
No. (unique) reflections	459893	466658
R_{work} / R_{free}	18.5 / 21.2	17.2 / 19.5
No. atoms		
Protein	33680	33647
Ligand	n/a	88
Solvent/ions	3622	4061
B-factors		
Protein	30.4	28.9
Ligand	n/a	20.3
Solvent/ions	36.2	37.3
R.m.s deviations		
Bond lengths (Å)	0.011	0.011
Bond angles (º)	1.48	1.47

*Values in parentheses denote highest resolution shell.

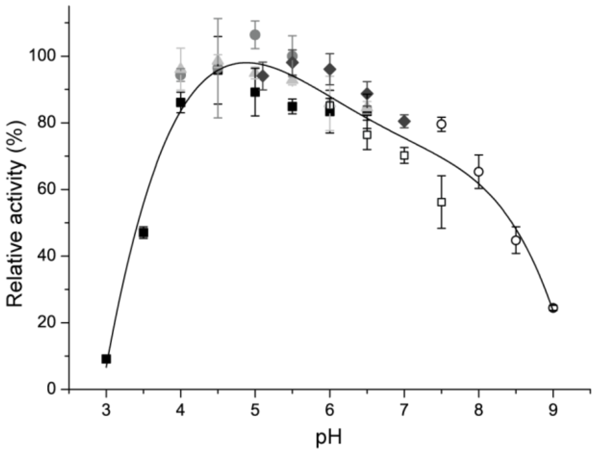

Figure S1: pH-rate profile of *Cj*Bgl35A. The following buffers were used at 50 mM: citrate (filled squares), acetate (filled circles), succinate (triangles), MES (diamonds), phosphate (empty squares), and glycylglycine (empty circles). The line is an arbitrary smooth curve through the data to guide the eye.

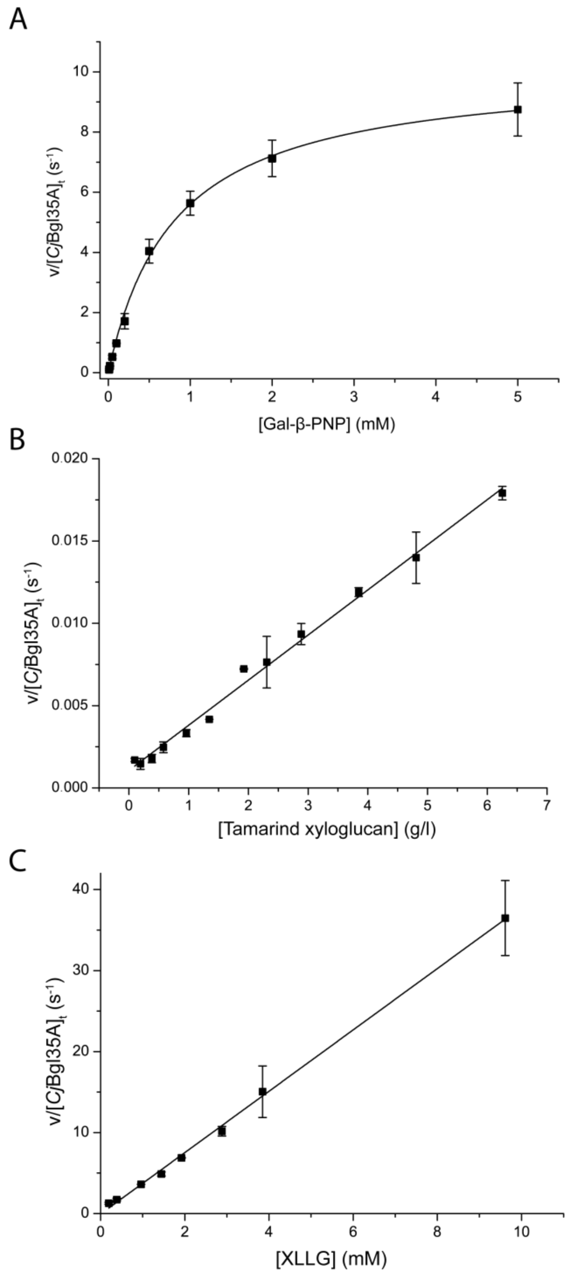

Figure S2: Kinetic analysis of the hydrolysis of galactosides by *Cj*Bgl35A. (A) Gal-β-PNP, (B) tamarind xyloglucan, and (C) XLLG. The amount of available galactose, used for calculating the velocity and the k_{cat}/K_m value for tamarind xyloglucan, was calculated from the ratio of galactose present in the xyloglucan preparation (16 %, as reported by the manufacturer).

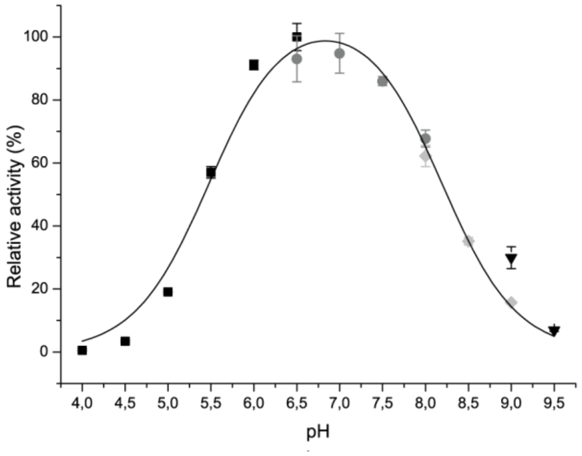

Figure S3: pH-rate profile of *Cj*Afc95A. The following buffers were used at 50 mM: citrate (squares), circles (sodium phosphate), glycylglycine (diamonds) and glycine (triangles).

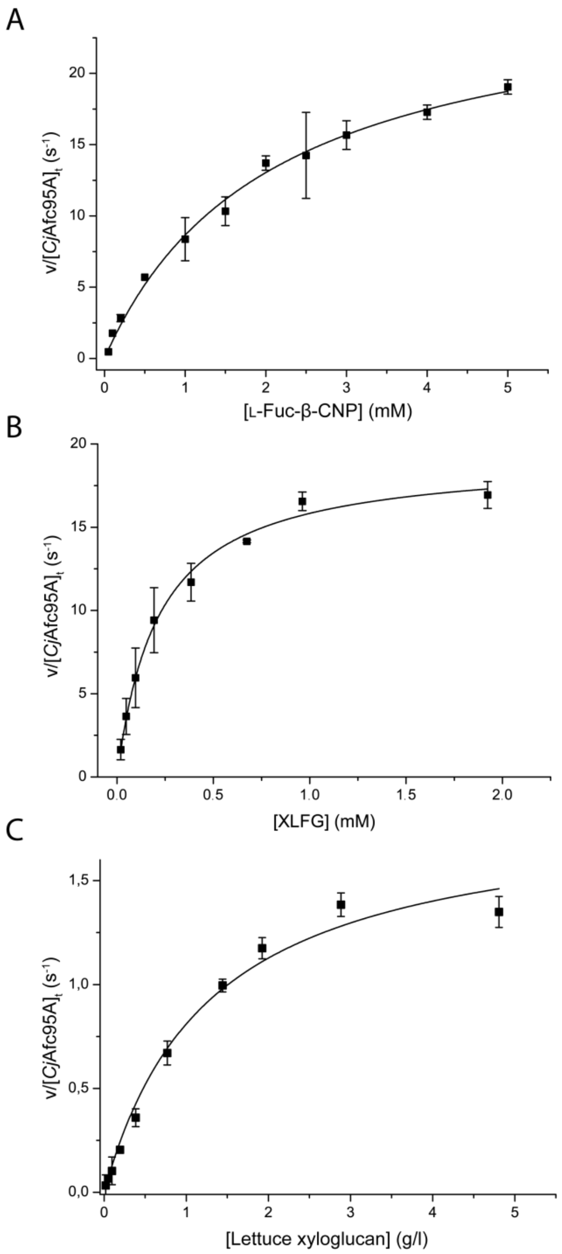

Figure S4: Kinetic analysis of the hydrolysis of fucosides by *Cj*Afc95A. (A) L-Fuc-α-CNP, (B) XLFG, and (C) lettuce XyG.

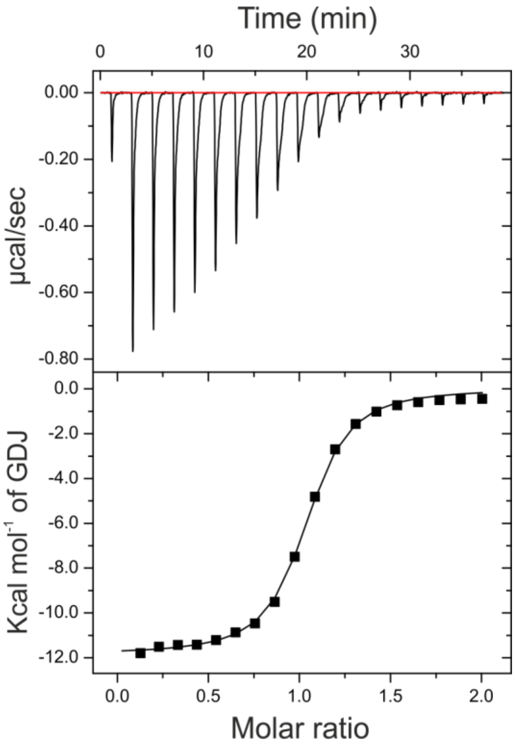

Figure S5: ITC thermogram of GDJ binding to Bgl35A. GDJ (390 μM) binds to Bgl35A (38.5 μM) with an average molar ratio (N) of 1.01 ± 0.0054 and average K_d of 485 ± 42 nM.

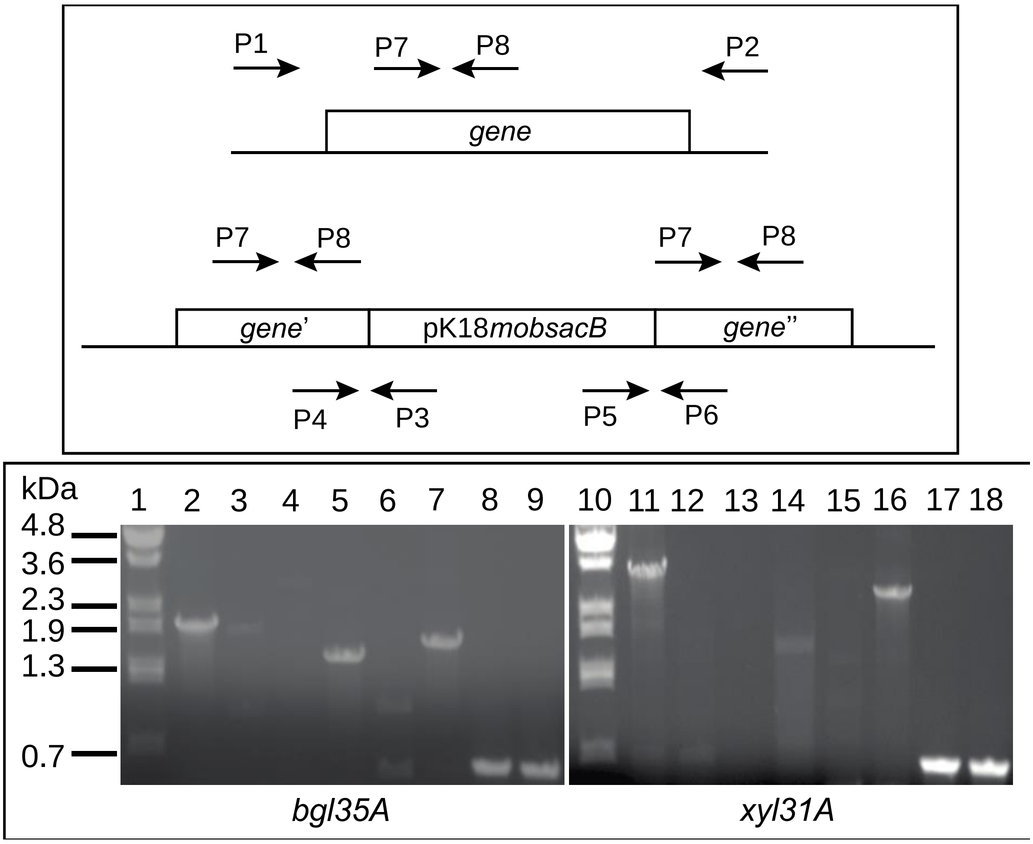

Figure S6: PCR confirmation of *bgl35A* and *xyl31A* mutations in *C. japonicus*. Overnight cultures of wild type and mutant strains were used as template for PCR, which were then run on 1% agarose gels, stained with ethidium bromide, and visualized on a BioRad GelDoc XR+ system. Top panel: Schematic for suicide plasmid pK18*mobsacB* insertion into a target gene and locations of the confirmatory primers used in the PCR series. Bottom panel: Gel photo confirming insertional inactivation of the *bgl35A* (left) and *xyl31A* (right) genes. In each case, the primers (P1, P2, etc.) are specific to either *bgl35A* or *xyl35A*, as indicated in Supplemental Table S1. **Lane 1**, λ *BstE*II DNA ladder; **Lane 2**, PCR of *bgl35A* region from wild type *C. japonicus* with primers P1 and P2; **Lane 3** PCR of *bgl35A* region from *bgl35A* mutant with primers P1 and P2; **Lane 4**, PCR from wild type with primers P3 and P4; **Lane 5**, PCR from the *bgl35A* mutant with primers P3 and P4; **Lane 6**, PCR from wild type with primers P5 and P6; **Lane 7**, PCR from the *bgl35A* mutant with primers P5 and P6; **Lane 8**, PCR of internal *bgl35A* region from wild type with primers P7 and P8; **Lane 9**, PCR of internal *bgl35A* region from the *bgl35A* mutant with primers P7 and P8; **Lane 10**, λ *BstE*II DNA ladder; **Lane 11**, PCR of *xyl31A* region from wild type *C. japonicus* with primers P1 and P2; **Lane 12,** PCR of *xyl31A* region from *xyl31A* mutant with primers P1 and P2; **Lane 13**, PCR from wild type with primers P3 and P4; **Lane 14**, PCR from the *xyl31A* mutant with primers P3 and P4; **Lane 15**, PCR from wild type with primers P5 and P6; **Lane 16**, PCR from the *xyl31A* mutant with primers P5 and P6; **Lane 17**, PCR of internal *xyl31A* region from wild type with primers P7 and P8; **Lane 18**, PCR of internal *xyl31A* region from the *xyl31A* mutant with primers P7 and P8.
